# EGF-Induced VEGF Exerts a PI3K-Dependent Positive Feedback on ERK and AKT through VEGFR2 in Hematological *In Vitro* Models

**DOI:** 10.1371/journal.pone.0165876

**Published:** 2016-11-02

**Authors:** Lilian Saryeddine, Kazem Zibara, Nouhad Kassem, Bassam Badran, Nabil El-Zein

**Affiliations:** 1 ER045, PRASE, EDST, Lebanese University, Beirut, Lebanon; 2 Laboratory of Cardiovascular Diseases and Stem Cells, Biology Department, Faculty of Sciences-I, Lebanese University, Beirut, Lebanon; 3 Biochemistry Department, Faculty of Sciences-I, Lebanese University, Beirut, Lebanon; Universite de Sherbrooke, CANADA

## Abstract

EGFR and VEGFR pathways play major roles in solid tumor growth and progression, however, little is known about these pathways in haematological tumors. This study investigated the crosstalk between EGFR and VEGFR2 signaling in two hematological *in vitro* models: THP1, a human monocytic leukemia, and Raji, a Burkitt’s lymphoma, cell lines. Results showed that both cell lines express EGFR and VEGFR2 and responded to EGF stimulation by activating EGFR, triggering VEGF production and phosphorylating ERK, AKT, and p38 very early, with a peak of expression at 10–20min. Blocking EGFR using Tyrphostin resulted in inhibiting EGFR induced activation of ERK, AKT, and p38. In addition, EGF stimulation caused a significant and immediate increase, within 1min, in pVEGFR2 in both cell lines, which peaked at ~5–10 min after treatment. Selective inhibition of VEGFR2 by DMH4, anti-VEGFR2 antibody or siRNA diminished EGF-induced pAKT and pERK, indicating a positive feedback exerted by EGFR-induced VEGF. Similarly, the specific PI3K inhibitor LY294002, suppressed AKT and ERK phosphorylation showing that VEGF feedback is PI3K-dependent. On the other hand, phosphorylation of p38, initiated by EGFR and independent of VEGF feedback, was diminished using PLC inhibitor U73122. Moreover, measurement of intracellular [Ca^2+^] and ROS following VEGFR2 inhibition and EGF treatment proved that VEGFR2 is not implicated in EGF-induced Ca^2+^ release whereas it boosts EGF-induced ROS production. Furthermore, a significant decrease in pAKT, pERK and p-p38 was shown following the addition of the ROS inhibitor NAC. These results contribute to the understanding of the crosstalk between EGFR and VEGFR in haematological malignancies and their possible combined blockade in therapy.

## Introduction

Growth factors and their receptors are essential for normal growth and development. However, their dysfunction causes cancer initiation and progression, making them attractive targets for anticancer therapy [1]. Among these factors are the epidermal growth factor (EGF) and its receptor (EGFR) and the vascular endothelial growth factor (VEGF) and its receptor (VEGFR), which constitute key elements in tumor growth and dissemination [2].

EGFR is a member of human EGF receptor (HER) family of tyrosine kinases whose dysregulated signaling is involved in many cancers of epithelial origin, accounting for 80% of all solid tumors [3]. EGFR overexpression or constitutive activation has been associated with increased tumor proliferation, survival, migration, and metastasis. Binding of EGF ligand to EGFR causes dimerization and auto-phosphorylation of the receptor, triggering a cascade of downstream signaling pathways such as Ras-MAPK, PI3K-Akt, and STAT [4]. The majority of solid cancers also overexpress VEGF, a potent stimulator of angiogenesis, whose receptor VEGFR2 plays a key role in transmitting signals for proliferation, differentiation, and migration of endothelial cells (ECs). VEGF also promotes microvascular hyperpermeability, which can both precede and accompany angiogenesis, favoring tumor stroma formation and tumor cell spreading [5]. Although acting primarily on vascular endothelium, VEGF produced by tumors operates in an autocrine loop on VEGF receptors expressed by tumor cells [6].

In a tumor hypoxic environment, stabilized hypoxia-inducible transcription factors (HIFs) bind to VEGF promoter and activate its transcription [7]. Interestingly, hypoxia-induced activation of HIF is accompanied by translational upregulation of EGFR and prolonged EGFR signaling [8]. Moreover, EGF and transforming growth factor-β (TGFβ), two potent EGFR ligands, have been shown to induce VEGF expression in cell culture models. Furthermore, different classes of EGFR inhibitors such as mAbs directed against the external ligand-binding site of the receptor, including cetuximab and panitumumab, in addition to small molecule tyrosine kinase inhibitors (TKIs) directed against the intracellular tyrosine kinase domain, like gefitinib, erlotinib and lapatinib, were able to attenuate VEGF expression *in vitro* and *in vivo [9]*. On the other hand, VEGF upregulation contribute to resistance to EGFR inhibition, independent of EGFR signaling [2]. Hence, VEGF signaling is up-regulated by EGFR expression whereas inhibition of VEGF-related pathways is thought to contribute to the mechanism of action for agents targeting the EGFR.

Although an increased association between VEGF and EGFR signaling pathways has been identified in various solid tumors, it is still poorly established in hematological malignancies. In fact, angiogenesis and its mediator, VEGF, were thought to minimally contribute to the pathogenesis of liquid tumors where EGFR has not been shown to be expressed. In contrary, elevated levels of VEGF and increased angiogenesis have been observed in most hematological malignancies and hence contribute to their pathogenesis [10]. Indeed, VEGF was found to trigger growth, survival, or migration of leukemia, lymphoma, and multiple myeloma cells through autocrine mechanisms [11, 12]. Recent studies demonstrated that human EGF-like receptor 2 (HER-2/Neu), structurally related to EGFR, is overexpressed in one-third of B-cell acute lymphoblastic leukemia (B-ALL) patients, who display resistance to conventional chemotherapies [13]. Moreover, another study demonstrated that one-third of human acute myeloid leukemia (AML) patients express EGFR, which is associated with poor clinical outcome. Furthermore, EGFR has been also detected in some leukemic cell lines [14]. Finally, malignant cells of most multiple myeloma patients overexpress a number of EGFRs and their ligands, which reinforces a role for this GF family in the pathophysiology of the disease [15].

In this study, we investigated the interaction of EGFR and VEGF-A pathways in the context of two hematological *in vitro* models: THP1, a human monocytic leukemia, and Raji, a Burkitt’s lymphoma, cell lines. We examined EGFR-induced VEGF-A production, VEGF-A feedback through VEGFR2, signaling pathways and cellular processes involved such as ERK, AKT, Ca^2+^ release, and ROS production.

## Materials and Methods

### Cell lines and culture conditions

THP-1 and Raji cell lines were obtained from the American Type Culture Collection (ATCC). Cells were maintained in Roswell Park Memorial Institute medium (RPMI; Lonza, Basel, Switzerland), supplemented with 10% Fetal Bovine Serum (FBS; Gibco, Life Technologies) and 1% Penicillin-Streptomycin (PS; Sigma-Aldrich, USA) and incubated at 37°C in 5% CO_2_.

### Reagents

EGF (Sigma) was used at 20 ng/ml, unless otherwise specified. The following inhibitors were used: EGFR tyrosine kinase inhibitor Tyrphostin (AG1478), VEGFR2 inhibitor Dimethylhydrazine (DMH4), also called 6-[4-[2-(4-Morpholinyl)ethoxy]phenyl]-3-phenylpyrazolo[1,5-a]pyrimidine, PI3K inhibitor 2-(4-Morpholinyl)-8-phenyl-1(4H)-benzopyran-4-one hydrochloride (LY294002), Phospholipase C inhibitor 1-[6-[[(17β)-3-Methoxyestra-1,3,5[10]-trien-17-yl)amino]hexyl]-1H-pyrrole-2,5-dione (U73122), ROS inhibitor N-acetyl cysteine (NAC). All Inhibitors were purchased from Sigma (USA).

### siRNA Studies

One day before siRNA transfection, cells were plated at 2x10^5^/mL in 12-well plates in DMEM cell growth media without antibiotics. Sense siRNAs targeting human VEGFR2 5'-CAAAUCUCAACGUGUCACU-3' (ON-TARGET plus SMART pool, catalogue no. L-003114-00-0005), and anti-sense VEGFR2 siRNA 5'-AGUGACACGUUGAGAUUUG-3' (catalogue no. D-001810-10-05) were purchased from Dharmacon. Each siRNA was used at 20 nM concentration and transfected into cells using the lipofectamine reagent according to manufacturer’s protocol (Invitrogen). Effects of siRNA on protein expression were assessed by western blot at 72h post transfection.

### RNA extraction

Total RNAs from cultured cells were isolated using TriPure kit according to manufacturer's instructions (Roche, USA). The quantity of RNA was measured using the Eppendorf Biophotometer Plus Spectrophotometer. RNA purity was assessed using the absorbance ratio of 260 to 280 nm, where a value of 1.8–2.0 indicated good quality RNA.

### RT-PCR

Reverse transcription was carried out on 1μg of RNA, using the qScript cDNA™ SuperMix (Quanta Biosciences, USA), according to manufacturer’s instructions. The resulting cDNA was then used for PCR using the following primers: VEGFR1, F: 5’- AAGAGAGCTTCCGTAAGGCG-3’, R: 5’-GCATCCTCTTCAGTTACGTCC-3’, VEGFR2, F: 5´-GGAAGCTCCTGAAGATCTGT-3´, R: 5´-GAGGATATTTCGTGCCGCGC-3´, VEGFR3, F: 5’-AGTCACACGTCATCGACACC-3’, R: 5’-CTTCCTGTTGACCAAGAGCG-3’, EGFR, F: 5´-AGGAGCTGCCCATGAGAAAT-3´, R: 5´-ATTGGGACAGCTTGGATCAC-3´, GAPDH, F: 5´-GTGTTCCTACCCCCAATGTGT-3´, R: 5´-ATTGTCATACCAGGAAATGAGC-3´. The PCR conditions were 95°C for 5min, then 40 cycles each of denaturation at 95°C for 1min, annealing at 60°C for 45s, extension at 72°C for 30s and a final extension step at 72°C for 20min. Results were analyzed by agarose gel electrophoresis. Negative control (water without DNA) was used to check for contamination.

### Protein extraction and quantification

Proteins from cultured cells were homogenized with RIPA buffer (50mM Tris, 150 mM NaCl, 1 mM EDTA, 0.5% NP40, and 0.1% sodium dodecyl sulfate [SDS]) containing protease and phosphatase inhibitors (Roche Applied Science, Germany). The lysates were incubated for 15min on ice followed by centrifugation at 11000 rpm for 15 min at 4°C and protein concentrations determined using Bradford Assay.

### Western blotting

Protein samples were mixed with loading buffer (Lamaelli and β-Mercapto-ethanol), heated at 95°C for 10min and then loaded into the wells of stacking gel and run until bromophenol blue reached the bottom of the gel. Gels were then transferred to PVDF membranes (Bio-Rad, Germany) at 4°C at 80–100 volts for 1h. The membrane was then blocked in 5% BSA, prepared in wash buffer (50 mM Tris-HCl, pH 8.8, 150 mM NaCl, and 0.05% Tween 20), for 2h at RT. Detection of the protein of interest was achieved by probing the membrane with the primary antibody of interest. Primary antibodies used were monoclonal anti-EGFR, p-EGFR, VEGFR2 (sc-6251), p-VEGFR2, VEGF-A (sc-152), ERK1, ERK1/2, p-ERK1/2, p38, p-p38, AKT, p-AKT. All antibodies were purchased from Santa Cruz and used at 1:1000 dilution. It’s worth noting that ERK1 is detected as 1 band on the western-blot whereas 2 bands corresponds to either ERK1/2 or p-ERK1/2. The phosphorylation sites recognized when using p-EGFR, p-VEGFR2, p-ERK, p-p38 and p-AKT corresponds to Tyr1068, Tyr1175, Tyr204, Tyr182, and Ser473; respectively. Actin antibody was used to ensure equal loading of samples (1:3,000 dilution). After several washes, the membrane was then incubated with horseradish peroxidase-conjugated anti-rabbit secondary antibody (diluted at 1:10,000) for 1h at RT. Protein bands were visualized using a chemiluminescent detection system (FluorChem E FE0324). Molecular weight markers enabled the determination of protein sizes. Intensity of bands was then determined by densitometry, using ImageJ software.

### ELISA

Conditioned media was employed to quantitatively measure VEGF-A using a sandwich enzymatic method with specific anti-VEGF-A antibodies (R&D Systems). The supernatant was collected and used for ELISA according to manufacturer's guidelines. Briefly, cells were grown to confluence in media supplemented with 10% FBS which was then replaced with serum-free medium. A total of 200 μl of cell supernatant were incubated with 50 μl of assay diluent for 2h at room temperature in a 96-well plate coated with a monoclonal antibody against VEGF165. After three washes, a conjugate consisting of a polyclonal VEGF antibody and horseradish peroxidase was incubated for 2 h at room temperature. After addition of a color reagent, absorbance was measured at 450 nm in a Thermo-Max microplate reader. For standardization, serial dilutions of recombinant human VEGF165 were assayed at the same time.

### Calcium measurement

THP-1 or Raji cells were loaded with the calcium-sensitive dye Fluo-3/AM and free intracellular calcium was determined with a fluorometer (LS50B fluorescence spectrometer, Perkin Elmer, USA). Calcium concentration was calculated using the equation: [Ca^2+^] = {(dF–Fmin) / (Fmax–dF)} * Kd where dF is the observed fluorescence, Fmin is the fluorescence at low Ca^2+^ (5 mM EGTA), Fmax is the fluorescence at high Ca^2+^ (8 μM ionomycin), and Kd being 390 nM for Fluo-3/AM. Values for Fmin and Fmax were obtained at the end of each experiment. Peak values were recorded and plateau phases calculated as the area under the curve, starting at the peak and up to 200s thereafter, and expressed as arbitrary units.

### Assessment of ROS production

This has been performed as previously described [16]. Briefly, THP-1 or Raji cell lines were incubated at 37°C for 20 min with 20 mM 2,7-dichlorofluorescein diacetate (DCFH-DA) (Molecular Probes, USA). After labeling, cells were treated, and production of ROS was then assessed every 10 min by measuring DCF emission at 525 nm; using a fluorometer (LS50B fluorescence spectrometer, Perkin Elmer, USA). Results were expressed as relative mean fluorescence intensity ±SEM.

### Statistical analysis

Results are expressed as individual data or as the mean±SEM. Statistical comparisons were performed using the Student's t-test in order to determine statistical significance. The *p* value was determined and values for p<0.05, p<0.001, p<0.0001 (*, **, ***; respectively) were considered significant. Microsoft Excel and GraphPad softwares were used to perform statistical analysis.

## Results

### THP1 and Raji cell lines express EGFR and VEGFR2

Recent studies demonstrated that EGFR is expressed in hematological malignancies [13]. Similarly, VEGFR2 was shown to be selectively expressed on endothelial cells, but is also found on certain leukemias and lymphomas [15]. In this study, EGFR and VEGFR2 were examined in two hematological *in vitro* models: THP1 and Raji, a human monocytic leukemia and a Burkitt’s lymphoma cell lines; respectively.

**Fig 1:**
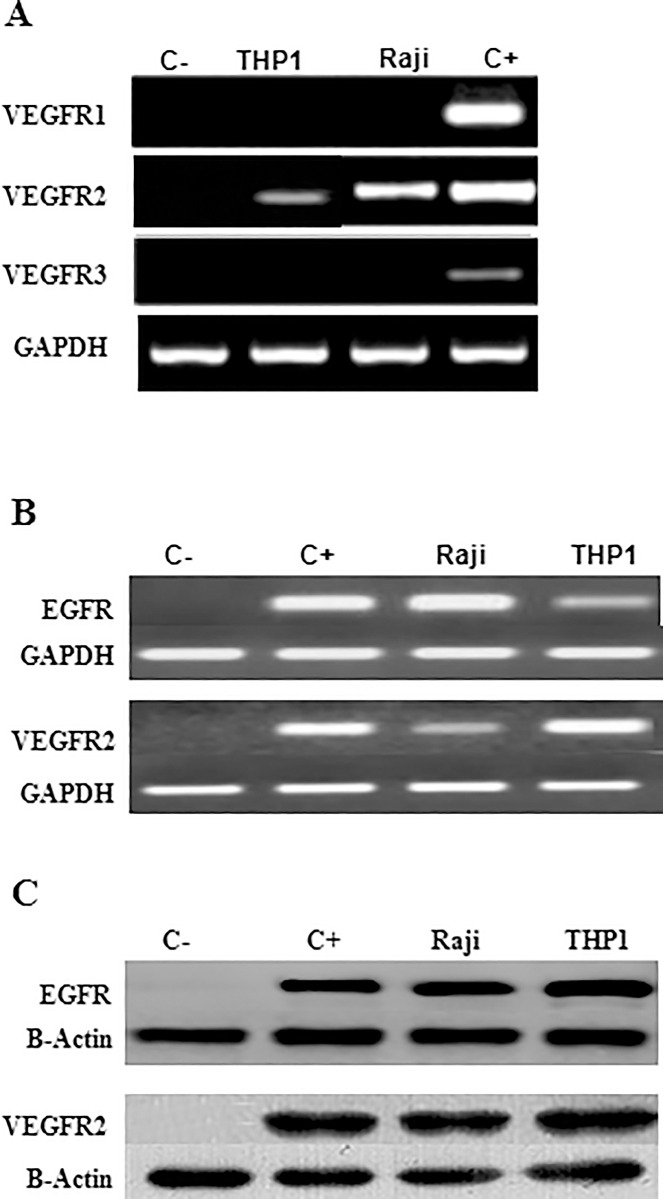
EGFR and VEGFR2 are expressed in THP1 and Raji cell lines. A total of 10^6^ cells/well were cultured. (A) VEGFR1 and VEGFR3 were found to be absent in both cell lines, using RT-PCR. (B) Cells were shown to express EGFR and VEGFR2 mRNA and (C) proteins using RT-PCR and western blot; respectively. Jurkat cell line was used as a negative control whereas monocytes were used as a positive control.

In order to study whether EGFR and VEGFR2 could induce any signaling pathway in THP1 and Raji cell lines, their expression was first explored at the transcriptional and translational levels. Indeed, both EGFR and VEGFR2 mRNA and protein levels were confirmed to be highly expressed in these cell lines, by RT-PCR and western blot, respectively (Fig 1B and 1C), similar to monocytes positive control, but unlike Jurkat cells which showed complete absence of expression, and considered as negative controls. It’s worth noting that VEGFR1 and VEGFR3 were found to be not expressed in those cell lines (Fig 1A).

### EGF activates p-EGFR which induces a significant increase in VEGF-A

**Fig 2:**
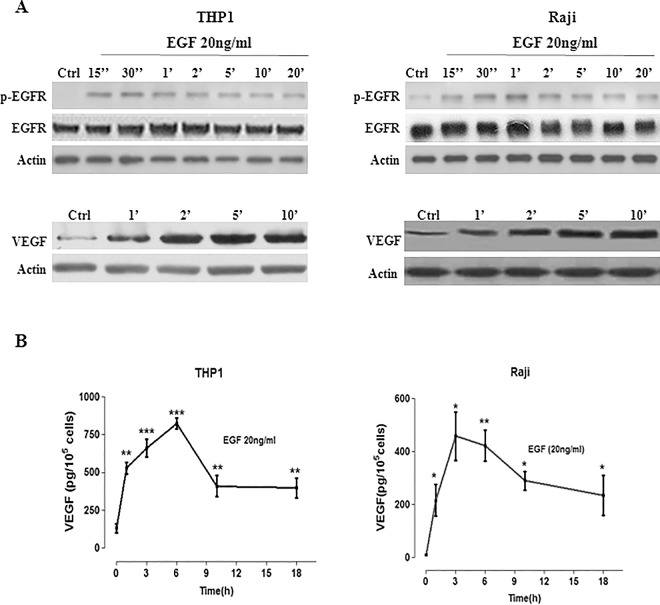
EGF activates p-EGFR which induces a significant increase in VEGF. A total of 10^6^ THP1 or Raji cells/well were cultured in the presence of 20ng/ml EGF at different time intervals. **(A)** Western blot analysis showing the effect of EGF on the levels of p-EGFR and those of VEGF at different times for THP1 (Left) and Raji (Right) cell lines. **(B)** ELISA analysis showing the effect of EGF on the levels of secreted VEGF at different times for THP1 (Left) and Raji (Right) cell lines. Results are representatives of four independent experiments (n = 4), for each time point and treatment condition. ELISA results are reported as the mean plus or minus the standard error of the mean (SEM). *, **, *** indicate p<0.05, p<0.001, p<0.0001; respectively.

To determine the effect of EGFR on VEGF expression, THP1 and Raji cells were treated with 20ng/ml of EGF for different time periods. Western-blot results showed a significant increase in p-EGFR levels after 15 seconds of EGF stimulation in the two cell lines (Fig 2A), indicating that EGFR is activated by ligand binding and is therefore functional. This activation of EGFR protein expression was maintained after 20 minutes of stimulation. In addition, a significant increase in intracellular VEGF-A protein levels was revealed after 1 minute of EGF stimulation (Fig 2A), which peaked after 5min, in both cell lines. Moreover, stimulation of Raji or THP1 cells by 20ng/ml EGF resulted in a significant increase in the levels of secreted VEGF-A, as measured by ELISA (Fig 2B). The secreted VEGF levels reached a maximum after 6 or 3 hours of EGF stimulation in THP1 or Raji cells; respectively (Fig 2B), demonstrating a time dependent VEGF induction by EGFR.

### EGFR-induced VEGF exerts a positive feedback on ERK and AKT signaling pathways

**Fig 3:**
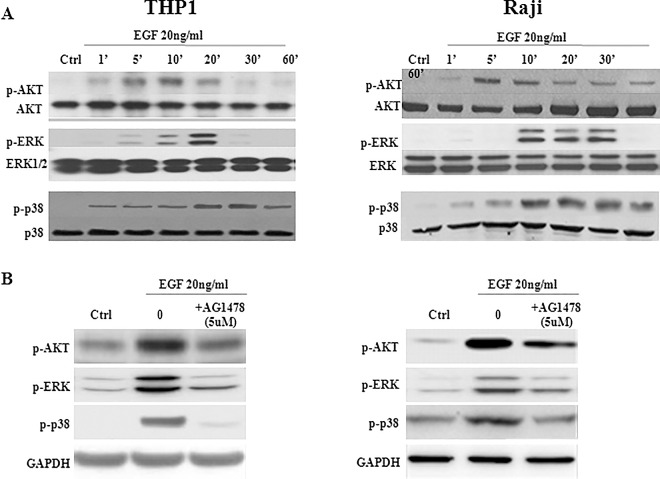
Effect of EGFR-induced VEGF on AKT, ERK and p38. A total of 10^6^ THP1 or Raji cells/well were cultured in the presence of 20ng/ml EGF and the phosphorylation levels of AKT, ERK and p38 were analyzed by Western blot. **(A)** EGF was treated for the indicated time intervals. Phosphorylation levels of p-AKT, p-ERK and p-p38 increased significantly after EGF stimulation in both THP1 (Left) and Raji (Right) cell lines. **(B)** In order to measure p-Akt, p-ERK and p-p38, EGFR was blocked using 5μM AG1478 inhibitor for 20min and cells were then stimulated by EGF at 20ng/ml for 10min, 20min, 30min or 5min, 10min, 20min in THP1 or Raji cell lines, respectively. The expression of pAKT, pERK, and p38 was found to be downregulated, using western-blot. It’s worth noting that ERK1/2 and p-ERK1/2 are detected as 2 bands on the western-blot corresponding to ERK1 and ERK2 or their phosphorylated isoforms. Results are representatives of four independent experiments (n = 4), for each time point and treatment condition.

Since MAPK and PI3K signaling cascades regulate VEGF expression, the activation of AKT, ERK, and p38 was examined after treatment with 20ng/ml EGF. Stimulation with EGF showed a significant and immediate increase in the phosphorylation levels of p-AKT, p-ERK and p-p38 in both cell lines (Fig 3A). Indeed, p-AKT levels peaked ~10 min after treatment in THP1 cells, whereas p-ERK and p-p38 reached their maximum levels after ~20 minutes. However, a more prominent and earlier response was observed in Raji cells where p-AKT peaked after ~5min of stimulation whereas only 10min were needed for p-ERK and p-p38 to reach their maximum levels (Fig 3A). In order to confirm our data, EGFR was blocked using the inhibitor Tyrphostin (AG1478) which resulted in a reduction in the expression of pERK, pAKT, and p-p38 (Fig 3B).

**Fig 4:**
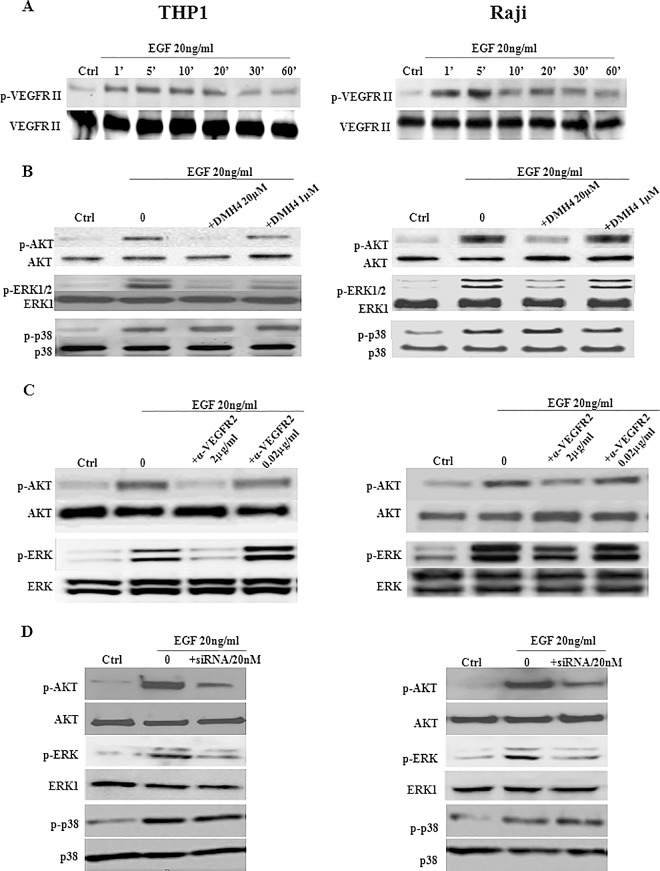
Effect of EGFR-induced VEGF on phosphorylation of VEGFR2, AKT and ERK. A total of 10^6^ THP1 or Raji cells/well were cultured in the presence of 20ng/ml EGF. **(A)** EGF stimulation increased very early the phosphorylation levels of p-VEGFR2 in THP1 (Left) or Raji (Right) cell lines, which peaked at ~5–10 min after treatment. **(B)** In order to measure p-Akt, p-ERK and p-p38, VEGFR2 was blocked using 20μM DMH4 inhibitor for 1h30min and cells were then stimulated by EGF at 20ng/ml for 10min, 20min, 30min or 5min, 10min, 20min in THP1 or Raji cell lines, respectively. Phosphorylation of AKT and ERK, but not p38, was diminished when the cell lines were pre-incubated with DMH4. **(C)** Similarly, phosphorylation of AKT and ERK, but not p38, was inhibited when cells were pre-incubated for 1h with 2μg/ml anti-VEGFR2 antibodies. **(D)** siRNA (20nM) against VEGFR2, left after transfection for 72h, caused a decrease in the phosphorylation levels of AKT and ERK, but not p38. It’s worth noting that ERK1/2 and p-ERK1/2 are detected as 2 bands on the western-blots corresponding to ERK1 and ERK2 or their phosphorylated isoforms. However, when ERK1 antibody is used, only 1 band appears on the blot. Results are representatives of four independent western blot experiments (n = 4).

On the other hand, stimulation with EGF showed a significant and immediate increase, within 1min, in the phosphorylation levels of p-VEGFR2 in both cell lines, which peaked at ~5–10 min after treatment (Fig 4A). However, pre-incubation of THP1 or Raji cells with 20μM DMH4, a selective VEGFR2 inhibitor, resulted in a significant decrease in EGF-induced phosphorylation of ERK and AKT, but not p38 (Fig 4B). This suggests that EGFR-induced VEGF will bind VEGFR2 in order to activate the downstream signaling pathways that drive its induction, in an autocrine feedback mechanism. Furthermore, similar inhibition of ERK and AKT phosphorylation, but not p38, was found using antibodies (Fig 4C) or siRNA (Fig 4D) directed against VEGFR2, which confirms an autocrine feedback mechanism in both cell lines through membranous, but not intracellular, VEGFR2.

### Activation of ERK and AKT is PI3K-dependent while PLC regulates p38

**Fig 5:**
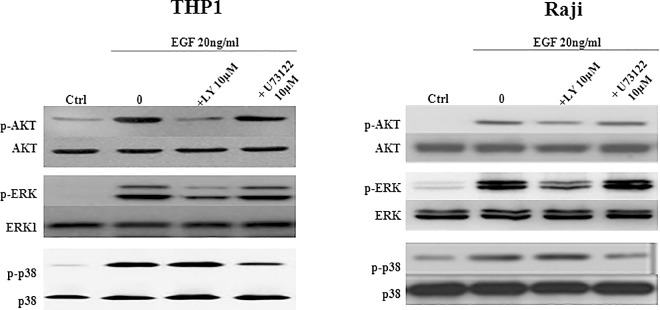
Activation of ERK and AKT is PI3K-dependent while PLC regulates p38. A total of 10^6^ THP1 or Raji cells/well were cultured and pretreated for 30min with 10μM of either LY294002 or U73122 inhibitor. In order to measure p-Akt, p-ERK and p-p38, cells were then treated with EGF at 20ng/ml for 10min, 20min, 30min or 5min, 10min, 20min in THP1 or Raji cell lines, respectively. Western blot analysis showing that EGF-induced phosphorylation of ERK and AKT was inhibited in the presence of LY294002 whereas that of p38 was inhibited by U73122. Results are representatives of three independent experiments (n = 3) for each treatment condition.

In addition to the canonical GRB2-SOS-RAS pathway, a PLC and a PI3K-mediated ERK activation exist. In order to determine which signaling pathway is involved in EGF-induced VEGF activation, THP1 or Raji cell lines were pretreated with a PI3K or PLC inhibitor, LY294002 or U73122; respectively, prior to EGF stimulation (Fig 5). Western blot analysis showed that PI3K inhibition by LY294002 resulted in a significant decrease in both p-ERK and p-AKT levels in both cell lines (Fig 5) whereas it did not have any effect on p-p38. On the other hand, the PLC inhibitor U73122 significantly reduced p-p38 levels in both cell lines while p-AKT and p-ERK levels were not affected. Therefore, we conclude that ERK and AKT activation depend on PI3K pathway while p38 activation depends on PLC pathway.

In summary, results of VEGFR2 inhibition showed that the greater part of ERK and AKT phosphorylation was triggered by VEGF feedback. Since ERK activation by VEGFR2 is not through GRB2-SOS-RAS and since VEGFR2 is strongly implicated in PI3K and PKC pathways, we may assume that PI3K pathway, through VEGFR2 activation initiated by EGFR, is responsible for ERK and AKT activation. On the other hand, EGFR seems to be the sole activator of p38 via PLC pathway.

### Effect of EGF and VEGF on intracellular [Ca2+] and ROS production

**Fig 6:**
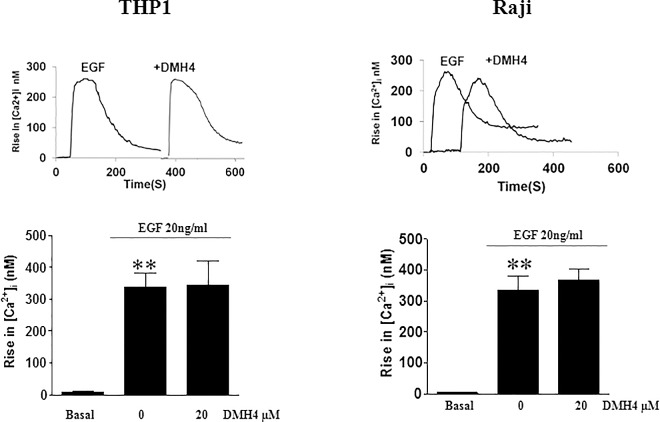
Measurement of intracellular calcium after EGF treatment, in the presence or absence of DMH4. THP1 or Raji cell lines were cultured as 10^6^ cells/well and incubated, or not, with 20**μ**M DMH4 for lh30min and then treated with 20ng/ml of EGF in a time-course at various time intervals. Fluorometry was then used in order to measure intracellualr calcium concentrations which were significantly and rapidly increased, in both cell lines, but were not affected by VEGFR2 inhibition using 20**μ**M DMH4. Results are representatives of three independent experiments (n = 3), for each time point and treatment condition, reported as the mean plus or minus the standard error of the mean. *, **, *** indicate p<0.05, p<0.001, p<0.0001; respectively.

Since p38 was found to be inhibited when PLC inhibitor U73122 was used, thus intracellular calcium is supposed to be released and therefore production of Calcium was measured within these cells. Intracellular Ca^2+^ and ROS are essential signaling mediators of growth factor receptors such as EGFR. The contribution of EGF and EGF-induced VEGF in the release of these intracellular messengers was examined in THP1 and Raji cell lines. Stimulation with 20ng/ml EGF for 1min resulted in a significant and rapid increase in intracellular calcium concentrations, in both cell lines. This surge of calcium was not affected by VEGFR2 inhibition using 20μM DMH4 (Fig 6).

**Fig 7:**
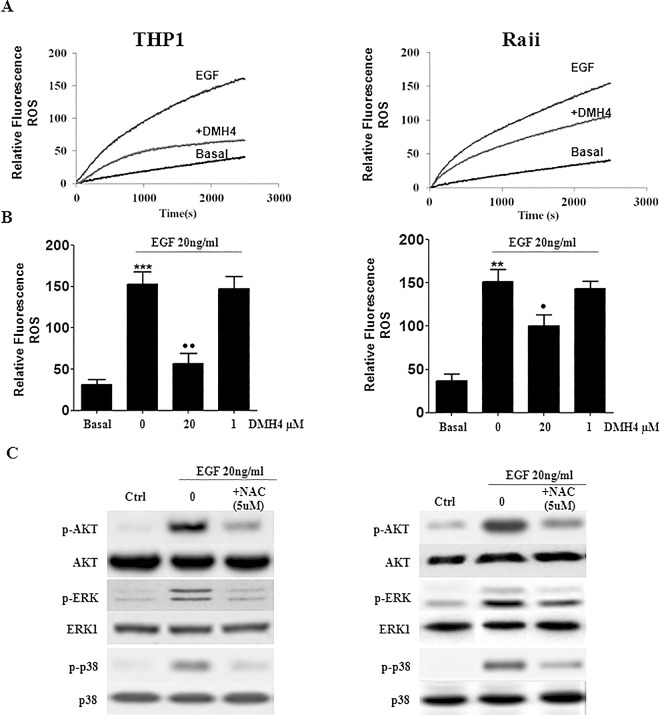
Measurement of ROS production after EGF treatment, in the presence or absence of DMH4 or NAC. THP1 or Raji cell lines were cultured as 10^6^ cells/well and pretreated for 1h30min with DMH4 (1μM or 20μM) or for 30min with N-acetylcysteine (NAC, 5μM) before treatment with 20ng/ml of EGF in a time-course. Following that, H₂O₂ production was measured using fluorometry. (A) Relative fluorescence curves at the basal level, after stimulation with EGF or after inhibition by DMH4 over a period of time. (B) Quantification of the relative fluorescence curve intensities. ROS production was significantly reduced by VEGFR2 inhibition, using 20μM DMH4. (C) In order to measure p-Akt, p-ERK and p-p38, cells were pretreated with 5μM of the ROS inhibitor N-acetylcysteine (NAC) for 30 min and then treated with EGF at 20ng/ml for 10min, 20min, 30min or 5min, 10min, 20min in THP1 or Raji cell lines, respectively. Western blot analysis showed a significant decrease in pAKT, pERK and p-p38. Therefore, VEGFR2 is responsible for EGF-induced ROS production. Results are representatives of three independent experiments (n = 3), reported as the mean plus or minus the standard error of the mean. *, **, *** indicate p<0.05, p<0.001, p<0.0001; respectively.

Since the increase in Calcium was found to be independent of VEGFR2, we investigated whether ROS, another messenger in cellular function, was solely dependent on EGFR or whether it’s dependent on VEGFR2. In contrast to what was obtained for Ca, EGF-induced VEGF resulted in ROS production which was significantly reduced by VEGFR2 inhibition, using 20μM of DMH4 (Fig 7A and 7B). Moreover, N-acetylcysteine (NAC) was used to inhibit ROS production followed by the measurement of pAKT, pERK and p-p38. Results showed a significant decrease in pAKT, pERK and p-p38 following the addition of the ROS inhibitor NAC (Fig 7C). These data confirm that EGF-induced phosphorylation of ERK, AKT and p38 is affected when ROS production is blocked. Therefore, VEGFR2 is not implicated in Ca^2+^ release whereas it’s responsible for EGF-induced ROS production.

## Discussion

The role of growth factors-driven signaling in the pathogenesis of human cancer has long been established for EGF, VEGF and their receptors EGFR and VEGFR [17]. Close relationship and signaling between these factors exist which result in survival and resistance mechanisms that prevent efficient targeted therapy [18]. In addition, it has been recently reported that VEGF and its receptor are crucial targets in angiogenesis [19] and that combination therapy using siRNAs enhance the antitumor therapy in xenografts [20]. Despite the extensive amount of research devoted to EGFR and VEGF in solid tumors, little is known about their involvement in liquid tumors. Recent findings, however, have shown their importance in the pathophysiology of hematological malignancies [3, 13]. This study investigated the molecular mechanisms of interaction between EGFR and VEGFR signaling pathways in two hematological *in vitro* models: THP1 and Raji, a human monocytic leukemia and a Burkitt’s lymphoma cell lines; respectively. The expressed EGFR and VEGFR2 showed important signaling interplay in the two cell lines for the following reasons. (1) Upon activation by EGF, p-EGFR induced a significant increase in VEGF-A, which exerted a positive feedback on ERK and AKT initiated by EGFR through VEGFR2. (2) VEGF-A feedback was PI3K dependent and VEGF also boosted EGF-induced ROS production. (3) On the other hand, in response to EGF, p38 phosphorylation was PLC dependent whereas it was independent of VEGFR2.

Conflicting reports exist about EGFR expression in hematological tumors [13, 21]. This study confirmed that both THP1 and Raji cell lines express EGFR at the mRNA and protein levels. This is in agreement with a recent study which detected EGFR transcripts in bone marrow and blood samples from acute monocytic leukemia (AML) M5 patients [14]. Moreover, we have demonstrated that VEGFR2 is detected at the transcriptional and translational levels in both cell lines. It’s important to note that VEGFR2 is expressed selectively, but not exclusively, on endothelial cells and that many tumors are known to express this receptor [6]. The first link between EGFR activity and VEGF expression was reported almost 20 years ago when it was shown that two EGFR ligands, EGF and TGFα, stimulated the expression of VEGF in glioma cells and in hyperproliferative keratinocytes [22]. Subsequent studies demonstrated that different classes of EGFR-targeting agents are able to attenuate VEGF expression [9]. In our study, EGF stimulated the phosphorylation of EGFR indicating responsive membranous EGFRs on both cell lines. EGF then induced the expression of VEGF-A, reflected by a significant increase of intracellular and secreted VEGF-A levels, in a time-dependent manner. Reaching a maximal VEGF-A response after 3-6h of stimulation may be explained by diffusion of the EGFR signal and/or possible negative feedback on VEGF induction. Lower extracellular VEGF levels found beyond this maximum indicated possible binding of secreted VEGF to VEGFR2 expressed on both THP1 and Raji cells.

VEGF expression is regulated by MAPK and PI3K signaling cascades and at least three different types of transcription factors: STAT3, Sp1 and HIFs [23]. This study investigated EGFR activation of two MAPKs, ERK and p38, in addition to the most important downstream target of PI3K, i.e. AKT. In response to EGF, phosphorylation of the three proteins was fast and peaked within minutes (~5–10 min) in THP1 and Raji cells. Moreover, inhibition of VEGFR2 by the selective inhibitor DMH4 attenuated EGF-induced ERK and AKT phosphorylation, indicating that the greater part of the activating signal is VEGFR2 dependent. VEGF has been identified as an autocrine factor in several solid tumors and leukemias expressing VEGF receptors, promoting their survival, proliferation, and metastasis [6]. Furthermore, anti-VEGFR2 antibody and siRNA were also able to inhibit ERK and AKT activation. As suggested above, released VEGF could bind VEGFR2 as an autocrine positive feedback mechanism. Indeed, EGF-induced VEGF production triggered the signaling via VEGFR2 resulting in the amplification of ERK and AKT pathways, known to upregulate VEGF expression. A relevant study demonstrated an autocrine feed-forward loop in NSCLC cells in which tumor-derived VEGF stimulated VEGF production via VEGFR2-dependent activation of PI3K/ mTOR [24]. Moreover, direct phosphorylation of HIF-1α and Sp1 by ERK1/2 has been shown to induce transcription of VEGF [9, 25, 26].

VEGFR2 is known to stimulate ERK via phosphorylation of PLC and subsequent activation of PKC, but not via GRB2-SOS-RAS as most other receptor tyrosine kinases (RTKs) [27]. Moreover, a non-canonical ERK activation via PI3K pathway also exists [28]. While neither ERK nor AKT phosphorylation were affected by PLC inhibition, PI3K inhibitor abolished most of the pERK and pAKT. Our results imply that the larger part of pERK, induced by VEGFR2 feedback, is PI3K dependent. The minor ERK activation, initially stimulated by EGFR, is probably through the canonical pathway since inhibition of PKC or PI3K non-canonical pathways was ineffective in bringing pERK to basal levels. VEGFR2 is strongly implicated in activation of the PI3K pathway. AKT inhibition, observed following VEGFR2 inhibition, also diminished EGF-induced AKT phosphorylation to basal levels. Indeed, our data confirm that AKT pathway is initiated by EGFR and enhanced by VEGF feedback. Two mechanisms have been proposed to illustrate a possible involvement of PI3K in ERK activation. The first is Ras-dependent involving PI3K lipid products that may promote association of adaptor molecules such as Shc, Grb2, and Gab1 with the plasma membrane. The second involves PI3K activation of p21-activated kinase (PAK) via Rac to promote stimulation of Raf and MEK leading to ERK activation [28]. Furthermore, it has also been reported that VEGF-C promotes angiogenesis by induction of COX-2 in leukemic cells via the VEGFR3/JNK/AP-1 pathway [29]. In contrast, our study demonstrated that a crosstalk exists between EGFR and VEGFR2 and that VEGF-A via VEGFR2/AKT/ERK pathway are implicated in THP1 and Raji cell lines along with the involvement of ROS and Ca^2+^.

On the other hand, p38 MAPK activation was not affected by VEGFR2 inhibition but was only dependent on EGFR. In agreement, p-p38 was diminished using PLC, but not PI3K, inhibitor. Additionally, EGF induced a surge in intracellular calcium concentrations independent of VEGFR2 feedback. Intracellular Ca^2+^ is an important signaling mediator of EGFR and an essential cofactor for PKC activation, whereby [Ca^2+^]i increase in response to EGF agrees with the fact that EGFR would activate p38 via PKC.

Additionally, although substantial amounts of ROS were present in unstimulated THP1 and Raji cells, EGF-induced VEGF promoted ROS production that was significantly reduced by VEGFR2 inhibition. Recent evidence indicates that ROS may function as intracellular messenger in receptor signaling pathways. The most important multienzyme complex involved in the generation of ROS during signal transduction pathways is a membrane-bound NADPH oxidase which elevates ROS within few minutes after cell stimulation [30]. In endothelial cells, VEGF stimulates ROS production (superoxide anion and H_2_O_2_) via activation of NADPH oxidases. ROS produced by NADPH oxidase then inactivates the protein tyrosine phosphatases which negatively regulate VEGFR2 [31]. It’s worth noting that it’s already established that platelet derived growth factor (PDGF)-induced ROS production requires the stimulation of PI3K to activate Rac1 which may mediate signaling between the PI3K product and the putative NADPH oxidase [30].

It’s important to note that the levels of p-Akt, p-ERK, p-p38 and ROS were partially reduced under the variable inhibitory conditions. This suggests that other compensatory signaling pathways or other cross-talks with other receptors might be involved or activated. This remaining residual activity could have been obtained through second messengers or receptors (e.g. PLC, GPCR, etc…). In conclusion, the last decade has witnessed the approval of monoclonal antibodies (mAbs) and small molecule tyrosine kinase inhibitors (TKIs) for targeting of oncogenic signaling pathways. Generally, the clinical activity of these agents has been less than expected, in part due to feedback loops and cross-talks between different signaling pathways, thus the interest of inhibiting multiple pathways. The extensive degree of EGFR-VEGF(R) pathway cross-talk in many solid tumors rendered them a promising target for Vandetanib (ZD6474), the dual kinase inhibitor of EGFR and VEGFR [32]. Our study has revealed the interplay between EGFR and VEGFR in THP1 and Raji cells at the level of signaling pathways involved in survival, proliferation, and angiogenesis. Further studies need to evaluate the therapeutic advantage of their combined blockade in these tumors.
